# Colonization by Extended-Spectrum β-Lactamase-Producing Enterobacterales and Bacteremia in Hematopoietic Stem Cell Transplant Recipients

**DOI:** 10.3390/antibiotics13050448

**Published:** 2024-05-15

**Authors:** Luiza Arcas Gonçalves, Beatriz Barbosa Anjos, Bruno Melo Tavares, Ana Paula Marchi, Marina Farrel Côrtes, Hermes Ryoiti Higashino, Bruna del Guerra de Carvalho Moraes, José Victor Bortolotto Bampi, Liliane Dantas Pinheiro, Fernanda de Souza Spadao, Vanderson Rocha, Thais Guimarães, Silvia Figueiredo Costa

**Affiliations:** 1 Departamento de Moléstias Infecciosas e Parasitárias, Hospital das Clínicas HCFMUSP, Faculdade de Medicina da Universidade de São Paulo, São Paulo 05403-000, Brazil; luiza.arcas.goncalves@gmail.com (L.A.G.); bampijvb@gmail.com (J.V.B.B.); thais.guimaraes@hc.fm.usp.br (T.G.); 2 Laboratório de Investigação Médica em Protozoologia, Bacteriologia e Resistência Antimicrobiana—LIM/49, Faculdade de Medicina FMUSP, Universidade de São Paulo, São Paulo 05403-000, Brazil; beatrizbdosanjos@gmail.com (B.B.A.); marchi.ap@gmail.com (A.P.M.); marinafarrel@yahoo.com.br (M.F.C.); brudelguerra@yahoo.com.br (B.d.G.d.C.M.); 3 Departamento de Controle de Infecção Hospitalar, Instituto Central, Moléstias Infecciosas e Parasitárias, Hospital das Clínicas HCFMUSP, Faculdade de Medicina da Universidade de São Paulo, São Paulo 05403-000, Brazil; bruno.mtavares@hc.fm.usp.br (B.M.T.); f.spadao@hc.fm.usp.br (F.d.S.S.); 4 Departamento de Hematologia, Hemoterapia e Terapia Celular, Hospital das Clínicas HCFMUSP, Faculdade de Medicina, Universidade de São Paulo, São Paulo 05403-000, Brazil; lilianedp@uol.com.br (L.D.P.); vanderson.rocha@hc.fm.usp.br (V.R.)

**Keywords:** hematopoietic stem cell transplant, colonization, bloodstream infection, extended-spectrum β-lactamase-producing Enterobacterales

## Abstract

Background: Assessing the risk of multidrug-resistant colonization and infections is pivotal for optimizing empirical therapy in hematopoietic stem cell transplants (HSCTs). Limited data exist on extended-spectrum β-lactamase-producing Enterobacterales (ESBL-E) colonization in this population. This study aimed to assess whether ESBL-E colonization constitutes a risk factor for ESBL-E bloodstream infection (BSI) and to evaluate ESBL-E colonization in HSCT recipients. Methods: A retrospective analysis of ESBL-E colonization and BSI in HSCT patients was conducted from August 2019 to June 2022. Weekly swabs were collected and cultured on chromogenic selective media, with PCR identifying the β-lactamase genes. Pulsed-field gel electrophoresis (PFGE) and whole-genome sequencing (WGS) assessed the colonizing strains’ similarities. Results: Of 222 evaluated HSCT patients, 59.45% were colonized by ESBL-E, with 48.4% at admission. The predominant β-lactamase genes were *bla*_TEM_ (52%) and *bla*_SHV_ (20%). PFGE analysis did not reveal predominant clusters in 26 *E. coli* and 15 *K. pneumoniae* strains. WGS identified ST16 and ST11 as the predominant sequence types among *K. pneumoniae*. Thirty-three patients developed thirty-five Enterobacterales-BSIs, with nine being third-generation cephalosporin-resistant. No association was found between ESBL-E colonization and ESBL-BSI (*p* = 0.087). Conclusions: Although the patients presented a high colonization rate of ESBL-E upon admission, no association between colonization and infection were found. Thus, it seems that ESBL screening is not a useful strategy to assess risk factors and guide therapy for ESBL-BSI in HSCT-patients.

## 1. Introduction

Bloodstream infections (BSIs) are a common complication following hematopoietic stem cell transplants (HSCTs), particularly during the pre-engraftment period, and are significantly correlated with high rates of morbidity and mortality, which can reach up to 45% [1]. HSCT recipients are notably susceptible to developing BSI due to chemotherapy-induced mucositis and neutropenia [2]. Over the past decades, there has been a shift in the etiological profiles of BSIs, with a decrease in the contribution of Gram-positive cocci and an increase in Gram-negative bacilli, along with a rise in the proportion of multidrug-resistant agents [1]. Extended-spectrum β-lactamase-producing Enterobacterales (ESBL-E) have assumed a prominent role in BSIs in recent years; nevertheless, they are not inherently linked to increased mortality. In this context, inadequate empirical therapy stands as the primary risk factor for mortality in bloodstream infections [3].

Identifying HSCT recipients at a high risk of developing a BSI caused by Enterobacterales carrying resistant genes can assist in optimizing empirical therapy, enhancing patient outcomes, and preventing the spread of resistance in this setting. The evaluation of colonization by ESBL-E and its association with the risk of developing a BSI by the same strain holds significant importance in the management of these patients.

The objective of this study was to assess whether ESBL-E colonization constitutes a risk factor for ESBL-E BSI in HSCT patients. Furthermore, this study aimed to evaluate ESBL-E colonization in patients undergoing HSCTs, exploring the clonality of colonization strains and identifying the most prevalent resistance genes.

## 2. Results

A total of 222 patients who underwent HSCTs were evaluated during the study period. Most of these patients were male (61.7%), with a median age of 47.5 years. The most common underlying disease was multiple myeloma (n = 56; 25.2%), followed by Hodgkin’s lymphoma. Regarding the type of transplant, the majority were autologous (n = 149; 67.1%). Among the allogeneic transplants (n = 73), haploidentical transplants were the most frequently performed (n = 38; 52%). The clinical and demographic information of the patients is described in Table 1.

Among the evaluated patients, 59.45% (132/222) were colonized by ESBL-E. Of these 132 patients identified as colonized by ChromID ESBL agar, 65 tested positive for ESBL-producing strains through disk diffusion testing. Among these, a total of 138 strains were identified. *Escherichia coli* emerged as the predominant isolate, comprising 45.95% (n = 68) of the total isolates, followed by *Klebsiella pneumoniae* (n = 35) and *Proteus mirabilis* (n = 9), accounting for 23.65% and 6.08% of the isolates, respectively. Other agents identified included *Enterobacter cloacae*, *Klebsiella oxytoca*, *Morganella morganii*, *Klebsiella variicola*, *Serratia marcescens*, *Raoultella ornithinolytica*, and *Citrobacter farmeri*, each represented by a smaller number of isolates.

Of the colonized patients, 64 out of 132 (48.48%) were identified as carriers upon admission, while the remaining cases were detected during hospitalization, with a median of 20 days (range: 7–84 days). Strains from 31 patients were assessed for resistance genes, with 6 samples from 2019, 19 from 2020, 4 from 2021, and 2 from 2022, totaling 56 strains evaluated, of which 32 were *K. pneumoniae* and 24 were *E. coli*. The *bla*_TEM_ gene was found in 71.8% of *K. pneumoniae* isolates and 41.6% of *E. coli* isolates. The *bla*_SHV_ gene was detected in 34.4% of *K. pneumoniae* isolates and 4.2% of *E. coli* isolates. The *bla*_CTX-M-2_ gene was present in 20.8% of the tested *E. coli* isolates. Additionally, the *bla*_CTX-M-8_ gene was found in 16.6% of the evaluated *E. coli* isolates and 3.1% of the *K. pneumoniae* isolates. Furthermore, 28.1% of the *K. pneumoniae* isolates carried both the *bla*_SHV_ and *bla_TEM_* genes, while 8.3% of the *E. coli* isolates carried the *bla*_CTX-M-8_ and *bla*_TEM_ genes. Additionally, 4.1% of the *E. coli* isolates of the same species possessed the *bla*_CTX-M-8_, *bla_TEM_*, and *bla*_SHV_ genes.

**Figure 1:**
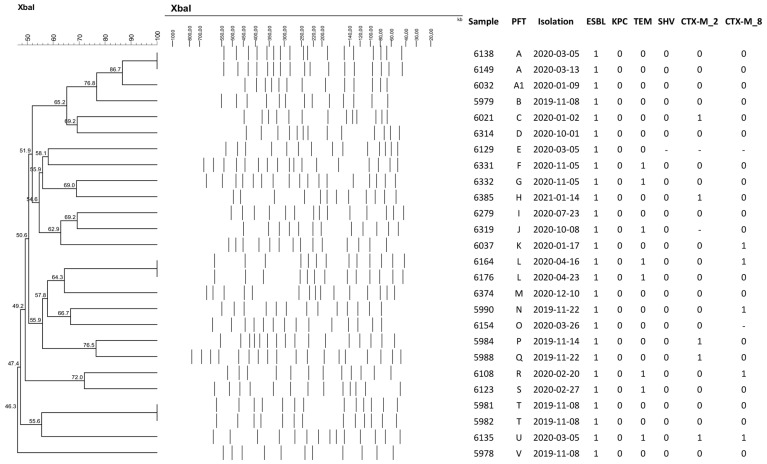
Dendrogram of the *Escherichia coli* samples identified in HSCT patients.

**Figure 2:**
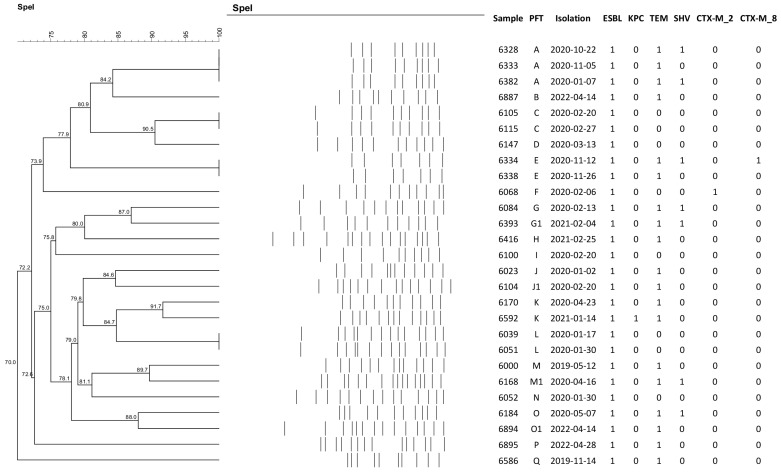
Dendrogram of the *Klebsiella pneumoniae* samples identified in HSCT patients.

Pulsed-field gel electrophoresis (PFGE) analysis of the 26 *E. coli* strains revealed 23 different pulsotype patterns, with no predominant cluster among the isolates. The identical pulsotype patterns identified were from samples from the same patients, as illustrated in the dendrogram of Figure 1. Additionally, 15 ESBL-producing *K. pneumoniae* strains were analyzed, revealing 12 different clones. One clone was identified in three distinct patients, with an interval of two to twelve weeks in relation to the initial isolate. The dendrograms of the *E. coli* and *K. pneumoniae* samples are illustrated below in Figure 1 and Figure 2, respectively.

During this period, 35 BSIs caused by Enterobacterales were identified, with 21 being attributed to *E. coli* and 14 to *K. pneumoniae*, in 33 patients (14.8%). Among these BSI cases, only nine were caused by pathogens resistant to third-generation cephalosporins (two by *E. coli* and seven by *K. pneumoniae*). All *K. pneumoniae* resistant to third-generation cephalosporins were also resistant to carbapenems.

During this period, urinary tract infections in patients undergoing HSCTs were also assessed. There were nine positive urine cultures, two of which were interpreted solely as colonization, both by carbapenem-resistant *K. pneumoniae*. Of these infections, two were caused by ceftriaxone-sensitive *E. coli*, one by ampicillin-sensitive *E. faecalis*, and four by *K. pneumoniae*, two of which were sensitive to ceftriaxone and two resistant to carbapenems. The incidence density of UTIs during various study intervals was as follows: from August to December 2019, there were no cases; from January to December 2020, there were 1.8 UTIs per 1000 patient days (n = 4 UTIs); in 2021, there were, again, no cases; and from January to June 2022, there were 0.3 UTIs per 1000 patient days (n = 1 UTI). Overall, during the entire study period encompassing 6789 patient days, we identified 5 UTIs, resulting in an incidence density of 0.7 UTIs per 1000 patient days.

The association between ESBL-E colonization and the occurrence of BSI caused by cephalosporin-resistant agents was evaluated, and regardless of the resistance profile, no association was found, as indicated in Table 2.

The association between Enterobacterales BSI and underlying diseases was evaluated using Fisher’s exact test, given the limited observations in certain groups. Underlying diseases included acute myeloid leukemia (AML), acute lymphoblastic leukemia (ALL), chronic lymphocytic leukemia (CLL), diffuse large B-cell lymphoma, Hodgkin’s lymphoma, multiple myeloma, myelodysplastic syndrome, aplastic anemia, other lymphomas, and other solid neoplasms. No statistically significant difference was observed (*p* = 0.203). Additionally, the association with the type of hematopoietic stem cell transplantation (HSCT)—autologous, related allogeneic, unrelated allogeneic, and haploidentical—was also evaluated, showing no association (*p* = 0.190).

**Figure 3:**
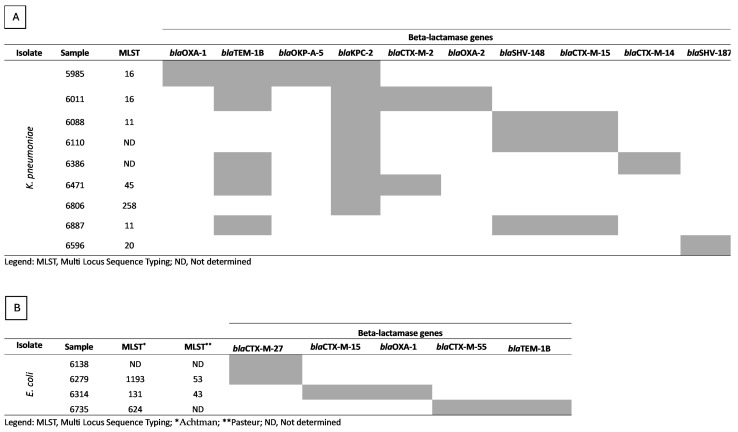
Genotypic characterization of *K. pneumoniae* and *E. coli* isolates resistant to beta-lactamase. (**A**) *K. pneumoniae*; (**B**) *E. coli*.

In the whole-genome sequence analysis, in *K. pneumoniae*, five different STs (ST11, ST16, ST45, ST258, and ST20) were observed. WGS analysis was unable to determine the type of MLST in two isolates. Resistome analysis identified important genes related to Beta-lactamase resistance, comprising *bla*OXA-1 (n = 1), *bla*TEM-1B (n = 5), *bla*OKP-A-5 (n = 1), *bla*KPC-2 (n = 7), *bla*CTX-M-2 (n = 2), *bla*OXA-2 (n = 1), *bla*SHV-148 (n = 3), *bla*CTX-M-15 (n = 3), *bla*CTX-M-14 (n = 1), and *bla*SHV-187 (n = 1) (Figure 3).

In the *E. coli* isolates, two different MLST schemes were evaluated. Using the Achtman scheme, three different MLST schemes were identified (ST1193, ST131, and ST624) and two STs (ST53 and ST43) were identified by the Pasteur scheme. Here, in three isolates, one isolate in the Achtman scheme and two isolates in the Pasteur scheme, the WGS analysis was also unable to determine the type of MLST. Regarding the resistome analysis, Beta-lactamase genes *bla*CTX-M-27 (n = 2), *bla*CTX-M-15 (n = 1), *bla*OXA-1 (n = 1), *bla*CTX-M-55 (n = 1), and *bla*TEM-1B (n = 1) were identified (Figure 3).

## 3. Discussion

In the present study, a high colonization rate of ESBL-E was found (59.45%), with approximately half of the cases present upon admission. The frequency identified was higher than previously reported in cohorts of patients undergoing HSCTs, with rates of 50% in Tunisia [4] (2020), 33% in India [5], 10% in the United States of America [6], and 7.6% in Italy [7].

Regarding the most frequent beta-lactamase gene, *bla*_TEM_ was identified in both *K. pneumoniae* and *E. coli*. The second most common beta-lactamase genes were *bla*_SHV_ in *K. pneumoniae* and *bla*_CTX-M-2_ in *E. coli*. The CTX-M gene has been the most frequently reported in previously published studies involving HSCT recipients, with frequencies ranging from 91% to 55% [6,8,9,10,11]. In a national assessment, *bla*_TEM_ was identified as the most prevalent beta-lactamase gene in both *E. coli* and *K. pneumoniae*, followed by *bla*_CTX-M_, with bla_SHV_ found in a smaller proportion of isolates [12]. Data from Brazil are scarce. Brazilian study found a higher frequency of *bla*_CTX-M_, specifically CTX-M-2, in the evaluation of 65 strains of cephalosporin-resistant *K. pneumoniae* [13]. In the present study, the proportion of isolates carrying two or more beta-lactamase genes was 12.5% for *E. coli* and 31.2% for *K. pneumoniae*, which was lower than what was found in a previous national assessment [12].

Approximately half of the colonized patients in the sample tested positive in surveillance swabs during the hospitalization period, with a median of 20 days for the length of stay. Approximately 43% of initially negative patients were colonized within the hospital setting. A German study that assessed colonization by ESBL-E in high-risk patients with hematological malignancies found a similar proportion of nosocomial colonization of around 43.3% [14]. Despite significant nosocomial colonization, the analysis of the PFGE patterns of the ESBL-E colonization strains revealed different pulsotype patterns, except for one clone identified in three distinct patients, with an interval of two to twelve weeks, which may indicate potential cross-transmission. The identification of colonization post-hospitalization does not necessarily imply intrahospital transmission; it could result from an increased bacterial density under the selective pressure exerted by antimicrobial treatment. However, an assessment of plasmid transmission between isolates was not conducted.

In the present study, we found an incidence of BSI by Enterobacterales of 14.8%, consistent with other cohorts of patients undergoing HSCTs, ranging from 8.6% to 22.7% [3]. Of all BSIs during the study period, nine were caused by pathogens resistant to third-generation cephalosporins. ESBL-E has gained increased significance as a causative agent of BSI in patients undergoing HSCTs in recent decades, particularly since the second decade of the 2000s [1]. However, a recent study at our center demonstrated a reduction in the contribution of ESBL-E to BSI cases starting in 2019, coinciding with the discontinuation of antimicrobial prophylaxis with fluoroquinolones during febrile neutropenia [15]. It is hypothesized that the use of prophylaxis may be associated with the selection of more resistant Gram-negative bacteria, resulting in bloodstream infections caused by more resistant strains.

In our study, no association was found between ESBL-E colonization and bloodstream infection caused by cephalosporin-resistant agents. The association between colonization and BSI by ESBL-E has been observed in cohorts of patients with hematological malignancy [10,16,17], corroborated by a meta-analysis published in 2016 [10,16,17]. In the context of HSCTs, a similar association was observed in American [6], Italian [7], and Brazilian [18] studies, all within the context of levofloxacin prophylaxis use.

In a study by Satlin and colleagues [6], colonization by ESBL-E was identified as a risk factor for BSI by the same agent, with more than 95% genetic similarity observed by PFGE between the colonizing strain and the one causing bloodstream infection. All strains were resistant to levofloxacin. It is hypothesized that levofloxacin prophylaxis eradicated other enteric bacteria, leading to the dominance of levofloxacin-resistant ESBL-E in the intestines. This state, in turn, resulted in the translocation of ESBL-E into the bloodstream during chemotherapy-induced neutropenia and mucositis. In pediatric HSCT cohorts without the use of prophylactic quinolones, no association was found between colonization and BSI by ESBL-E [5,19], a context similar to that of the current study.

The abundance of intestinal colonization by Gram-negative bacteria has been associated with a high predictive value for BSI in the context of allogeneic HSCTs [20]. The use of fluoroquinolone prophylaxis alters the abundance of Gram-negative bacteria within the intestinal tract, leading to a higher incidence of resistance in breakthrough infections. The significance of colonization by ESBL-E may be greater in the context of prophylaxis use, as it influences the selection of predominant strains in the gastrointestinal tract, favoring bloodstream infections by more resistant agents. Without the use of quinolones, even if ESBL-E is present, it may not act as the predominant strain, reducing the association of colonization with BSI.

Data regarding *E. coli* and *K. pneumoniae* lineages in HCST patients are scarce and have been focused on multidrug-resistant strains. The STs found among the *K. pneumoniae* at our hospital belonged to the clonal group 258 (CG258), which is considered a high-risk lineage that is often multi-resistant and spreads mainly in the hospital environment. CG258 is the most prevalent lineage in Brazilian hospitals [21,22]. The *E. coli* samples belonged to ST1193, ST131, and ST624. ST131 is the most frequent lineage, and ST1193 is the second most frequent lineage among cephalosporin-resistant *E. coli* isolates in the world. Both clones have played crucial roles in the worldwide spread of MDR *E. coli* [23]. *E. coli*-ST131 has been described in Brazil as causing urinary tract infections [24].

## 4. Materials and Methods

### 4.1. Study Design and Setting

From August 2019 to June 2022, we conducted a prospective evaluation of perineal and rectal swabs collected weekly from patients undergoing HSCTs at the Instituto Central do Hospital das Clinicas da Faculdade de Medicina da Universidade de São Paulo (IC-HCFMUSP), Brazil. The IC-HCFMUSP is a tertiary hospital with 665 beds, 103 of which are intensive care unit (ICU) beds. The hematopoietic stem cell transplantation unit consists of 16 individual beds, including 4 with positive pressure, where approximately 90 transplants, both autologous and allogeneic, are performed each year. Since January 2019, patients no longer receive antibacterial prophylaxis following a change in the institutional protocol, prompted by the increased antimicrobial resistance in Gram-negative bacteria causing BSIs in HSCT patients. Instead, they have received standard care for preventing healthcare-associated infections, with high hand hygiene compliance rates, along with an alcohol consumption of 43.3 mL/patient day over the past 5 years.

### 4.2. Microbiology

The isolates were preserved in the strain repository located at the Medical Research Laboratory (LIM 49) at the Instituto de Medicina Tropical, Universidade de Sao Paulo. The swabs collected were cultured on ChromID ESBL agar, a selective medium manufactured by Biomèriux (Lyon, France), specifically designed for the detection of ESBL-producing bacteria. Additionally, the isolates were cultured on MacConkey agar and incubated at 37 °C for 18 to 24 h. To identify the isolates, matrix-assisted laser desorption/ionization time-of-flight mass spectrometry (MALDI-TOF MS) with a Microflex mass spectrometer manufactured by Bruker Daltonics in Bremen, Germany, was employed. For the determination of extended-spectrum beta-lactamase (ESBL) production, the disk diffusion method standardized by the Clinical and Laboratory Standards Institute (CLSI, 2021) [25] was used. This method involved using commercial POLISENSIDISC-Brasil (DME^®^, São Paulo, Brazil) disks, containing a combination of five antibiotics: amoxicillin/clavulanic acid (AMC 30), aztreonam (ATM 30), ceftazidime (CAZ 30), ceftriaxone (CRO 30), and cefotaxime (CTX 30). The bacteria were cultured on Mueller–Hinton agar and incubated at 37 °C for 24 h. If a zone of growth inhibition formed around any of the antibiotics within the disk, the microorganism was classified as an ESBL producer. The results were interpreted according to the interpretative criteria recommended by the CLSI in 2021 [25]. Furthermore, polymerase chain reactions (PCR) targeting β-lactamase genes were performed after phenotypic ESBL testing to provide additional confirmation of the presence of ESBL genes in a subset of isolates, including samples from all years of this study [26]. The primers employed in the amplification reactions for beta-lactamase-encoding genes are detailed in Table 3.

During the same period, all BSIs and urinary tract infections caused by Enterobacterales were analyzed. A laboratory-confirmed BSI was defined as the growth of enterobacteria in one or more venipuncture blood cultures. Blood cultures were performed using the BD BACTEC (Becton Dickinson, Franklin Lakes, NJ, USA) automated system. Positively flagged blood culture bottles were cultured on blood agar and MacConkey agar, and isolated colonies were identified by MALDI-TOF. The Vitek-2 system was used for automated antimicrobial sensitivity testing according to the CLSI guidelines [25]. The clinical samples were not assessed for ESBL resistance genes; they were only identified as resistant to third-generation cephalosporins. Samples resistant to carbapenems underwent the modified Hodge test [25] to assess the carbapenemase production without genotypic evaluation.

All patients were individually assessed for their age, gender, underlying diseases, and the type of transplantation (autologous or allogeneic). In the case of allogeneic transplantation, the type of donor was specified, including matched-related donors, matched-unrelated donors, or haploidentical donors.

To understand the genetic relatedness between colonizing strains, pulsed-field gel electrophoresis (PFGE) was conducted using the enzyme *Spe*I for *K. pneumoniae* and *Xba*l for *E. coli* (Thermo Fisher Scientific, Waltham, MA, USA). The genetic profiles were analyzed using BioNumerics bioinformatics software v. 8.1 (Applied Maths, Sint-Martens-Latem, Belgium). This technique enabled the comparison of the genetic patterns of the bacteria, facilitating the identification of clonality among colonized patients, which is important for assessing potential cross-transmission events within the HSCT unit.

### 4.3. Whole-Genome Sequencing

Nine *K. pneumoniae* and five *E. coli* isolates were submitted for whole-genome sequencing (WGS) using the Ion S5 system, the Ion S5 sequencing kit, and the Ion 530 chip, following the manufacturer’s instructions (Thermo Fisher Scientific, Waltham, MA, USA). The genomes were mapped using the Galaxy platform [27], and the *Klebsiella pneumoniae* strain MGH78578 (GenBank accession number CP000647.1) and *Escherichia coli* strain K-12 substr. MG1655 (GenBank accession number NC_000913.3) were used as a reference for molecular characterization. Multi-locus sequence typing (MLST) and resistome analysis were performed using MLSTFinder 2.0 [28] and ResFinder 4.0 tolls [29], respectively. The whole-genome shotgun project of *K. pneumoniae* and *E. coli* isolates was deposited at the DDBJ/EMBL/GenBank under the Bioproject: PRJNA1100951.

### 4.4. Statistical Analysis

Categorical variables were presented as absolute numbers and percentages, while continuous variables were reported as medians and interquartile ranges (IQRs). The associations between colonization and BSI were assessed using a bivariate logistic regression model, as well as the association between underlying diseases and the type of hematopoietic stem cell transplantation (TCTH) and BSI. SPSS version 20 software (IBM, Armonk, NY, USA) was used for conducting these analyses.

## 5. Conclusions

Despite a considerable proportion of patients being colonized with ESBL-E, no association with BSI was identified. Additionally, no predominant clone of *K. pneumoniae* or *E. coli* was found among the colonized individuals. In the context of discontinuing antimicrobial prophylaxis, screening for ESBL-E colonization does not appear to significantly contribute to the identification of patients at a high risk for ESBL-E bloodstream infection.

## Figures and Tables

**Table 1:** Clinical and demographic characteristics of patients undergoing HSCTs between August 2019 and June 2022.

Characteristics	n (%) or Median [25th–5th Percentile]
Male	137 (61.7)
Age (years)	47.5 [30.25–57]
Underlying disease	
Multiple myeloma	56 (25.2)
Hodgkin’s lymphoma	40 (18.0)
Another lymphoma	35 (15.7)
Acute myeloid leukemia	26 (11.7)
Another hematological disease	23 (10.3)
Acute lymphocytic leukemia	19 (8.5)
Diffuse large B-cell lymphoma	8 (3.6)
Chronic myeloid leukemia	6 (2.7)
Aplastic anemia	6 (2.7)
Myelodysplastic syndrome	3 (1.3)
HSCT type	
Autologous	149 (67.1)
Allogeneic	73 (32.9)
Haploidentical	38 (52.0)
Matched-related donor	25 (34.2)
Matched-unrelated donor	10 (13.7)

**Table 2:** Association between colonization and risk of bacteremia by resistant Enterobacterales in HSCT patients.

	OR ^a^	OR CI ^b^	*p*-Value
***E. coli* resistant to 3rd-generation cephalosporins BSI**			
ESBL-E colonization (n/N = 2/132)	1.015	0.994–1.037	0.516
***K. pneumoniae* resistant to 3rd-generation cephalosporins BSI**			
ESBL-E colonization (n/N = 6/132)	4.238	0.501–35.818	0.246
***Enterobacterales* resistant to 3rd-generation cephalosporins BSI**			
ESBL-E colonization (n/N = 8/132)	5.742	0.706–46.731	0.087
***Enterobacterales* BSI**			
ESBL-E colonization (n/N = 23/132)	1.688	0.761–3.744	0.250

^a^ Odds ratio. ^b^ 95% confidence interval.

**Table 3:** Primers used for the detection of ESBL resistance genes.

Gene	Oligonucleotide Sequences (5′-3′)	Annealing Temperature	Size (bp)
*bla*_CTX-M-8_	CTX-M-8 F (240)—GATGAGACATCGCGTTAAG	52 °C	861
	CTX-M-8 R (241)—GGTGACGATTTTCGCGGCA		
*bla*_CTX-M-2_	CTXM-2 F GACTCAGAGCATTCGCCGC	55 °C	870
	CTXM-2 R TCAGAAACCGGGGTTACGA		
*bla*_TEM_	TEM CR F (233)—CGWGTCGCCCTTATTCCCT	55 °C	1066
	TEM R (235)—CCAAWGCTTAATCAGTGA		
*bla*_SHV_	SHV F CAGCGTGACATCATTCTGTG	55 °C	838
	SHV R TCTGCTTACCAGGCGCATTT		

## Data Availability

Data are contained within the article.
